# Dietary pesticide exposure and non-communicable diseases and mortality: a systematic review of prospective studies among adults

**DOI:** 10.1186/s12940-023-01020-8

**Published:** 2023-10-31

**Authors:** Julia Baudry, Pauline Rebouillat, Cécilia Samieri, Justine Berlivet, Emmanuelle Kesse-Guyot

**Affiliations:** 1 Université Sorbonne Paris Nord and Université Paris Cité, Inserm, INRAE, CNAM, Center of Research in Epidemiology and StatisticS (CRESS), Nutritional Epidemiology Research Team (EREN), Bobigny, F-93017 France; 2 Univ Bordeaux, Inserm, Bordeaux Population Health Research Center, UMR 1219, Bordeaux, France

**Keywords:** Pesticides, Health, Diet, Prospective study, Exposure, Adults

## Abstract

**Background:**

Research on the effect of pesticide exposure on health has been largely focused on occupational settings. Few reviews have synthesized the associations between dietary pesticide exposure and health outcomes in non-occupationally exposed adults.

**Objective:**

We aim to summarize the evidence regarding dietary pesticide exposure and non-communicable diseases (NCD) in adults, using a systematic review of prospective studies.

**Methods:**

Electronic and manual searches were performed until July 2023. The inclusion criteria were the following: 1) adults aged ≥ 18years, 2) (non)-randomized trials, prospective cohort studies, 3) dietary exposure to pesticides. A bias analysis was carried out using the Nutrition Evidence Systematic Review guidelines based on the Cochrane ROBINS-I.

**Results:**

A total of 52 studies were retrieved and 6 studies that met the above criteria were included. Studies were conducted either in France or in the United States. The studies investigated the risk of cancer (*n* = 3), diabetes (*n* = 1), cardiovascular diseases (*n* = 1), and mortality (*n* = 1). The quality of the studies varied with overall grades derived from the bias analysis ranging from low to moderate bias. The level of evidence was estimated as low for the risk of cancer while the grading was not assignable for other outcomes, as only one study per outcome was available.

**Conclusions:**

Although further research is warranted to examine more in depth the relationships between low-dose chronic exposure to pesticides through diet and NCD outcomes in non-occupationally-exposed adults, studies suggest a possible role of exposure to dietary pesticide on health. Standardized methodological guidelines should also be proposed to allow for comparison across studies.

**Supplementary Information:**

The online version contains supplementary material available at 10.1186/s12940-023-01020-8.

## Introduction

Cancer, diabetes, cardiovascular and chronic respiratory diseases are non-communicable diseases (NCD) responsible for 41 million deaths per year [1]. They account for over 80% of all premature NCD-related deaths worldwide [1]. Environment is a major determinant of health and according to the World Health Organization (WHO), known chemical substances (including pesticides) are responsible for 25% of chronic diseases worldwide [2].

Identifying possible levers for reducing exposure to pesticides would enable the development of effective public health strategies. In particular, minimizing exposure to active substances which are currently in use could help prevent NCD [3].

Agricultural pesticides are chemical substances widely used in agriculture to enhance food production, to which individuals are chronically exposed from both occupation (e.g. agricultural workers) and everyday life through air, dust, food/drink (e.g. general population).

The most common pathways of pesticide exposure include the cutaneous, digestive and respiratory routes. The cutaneous route is the main route of exposure in the workplace (i.e. among farmers, agricultural workers, manufacturers, and handlers of these substances). Exposure through the respiratory route concerns certain specific professional practices in closed environments. In the general population, the dietary route is considered the main route of exposure by the WHO, through the intake of contaminated food or drinks [4].

Experimental studies have documented a number of mechanistic pathways through which pesticide exposure could affect health [5–7]. With regard to epidemiological evidence, exposure to pesticides has been associated with increased risk of different pathologies such as non-Hodgkin’s lymphoma, multiple myeloma, prostate cancer, Parkinson’s disease as well as cognitive disorders and respiratory diseases [8–11].

Apart from the health burden, the massive use of pesticides could represent a significant social burden. Thus, a recent study estimated that the social costs attributable to synthetic pesticide use amounted to 372 million euros in 2017 in France, of which 48.5 million euros were related to health costs [12].

However, original research and review studies on the impact of pesticides on human health generally focus on occupational settings, without considering the general population for which diet is the main source of exposure.

Hence, this systematic review aims to synthesize the evidence, from prospective studies, concerning the associations between dietary exposure to pesticides and diet-related NCD and mortality caused by NCD in adults.

## Methods

We systematically reviewed prospective studies aimed at estimating the associations between dietary exposure to pesticides and NCD among adults. The methodology for conducting systematic review in nutrition and public health developed by the United States Department of Agriculture (USDA)’s Nutrition Evidence Systematic Review (NESR) team [13] was used. This systematic review was planned and conducted in accordance with the standards of PRISMA-2020 [14].

### Search strategy & eligibility criteria

An electronic search of articles was conducted using (MEDLINE) (via PubMed) until July 2023 with no restriction to calendar date. A PRISMA checklist is provided in Supplemental Table 1. The systematic review has been registered in Prospero (Number CRD42022383916).

The literature search was conducted by two authors (JBau, EK-G). Moreover, the reference lists from the identified articles were checked to search for further relevant studies.

The detailed query used in PubMed is presented in Supplemental Method 1.

### Inclusion and exclusion criteria

Studies were selected for the review if they met all the criteria, as described in Table 1.

**Table 1:** Inclusion and exclusion criteria among eligible studies

	Inclusion criteria	Exclusion criteria
**Type of study**	Original research published in peer-reviewed journal	No English-language article, review, meta-analysis, protocol study
**Study design**	Prospective	Retrospective, case–control, cross-sectional
**Populations**	Adults (≥ 18 years)	Children, agricultural workers
**Exposure**		
Source of exposures	Diet	Air, dust
Site of exposures	Dietary	Occupational, residential
Type of exposures	Chronic	Acute
Measurement of exposures	Consumption data coupled with contamination data	Biomonitoring
Pesticides	Pesticides, fungicides, insecticides, herbicides, pyrethroids, organophosphates, carbamates, glyphosate	Non-agricultural pesticides
**Outcomes**	NCD, NCD-related mortality	Infectious diseases, infertility disorders

*NCD* Non-communicable diseases

Human biomonitoring studies were excluded since they do not allow to identify the source of exposure. Regarding the outcome, the focus was on NCD and NCD-related mortality, i.e. non-infectious diseases which typically include the four main following types: cardiovascular diseases (CVD), diabetes, cancer and chronic respiratory diseases.

**Fig. 1:**
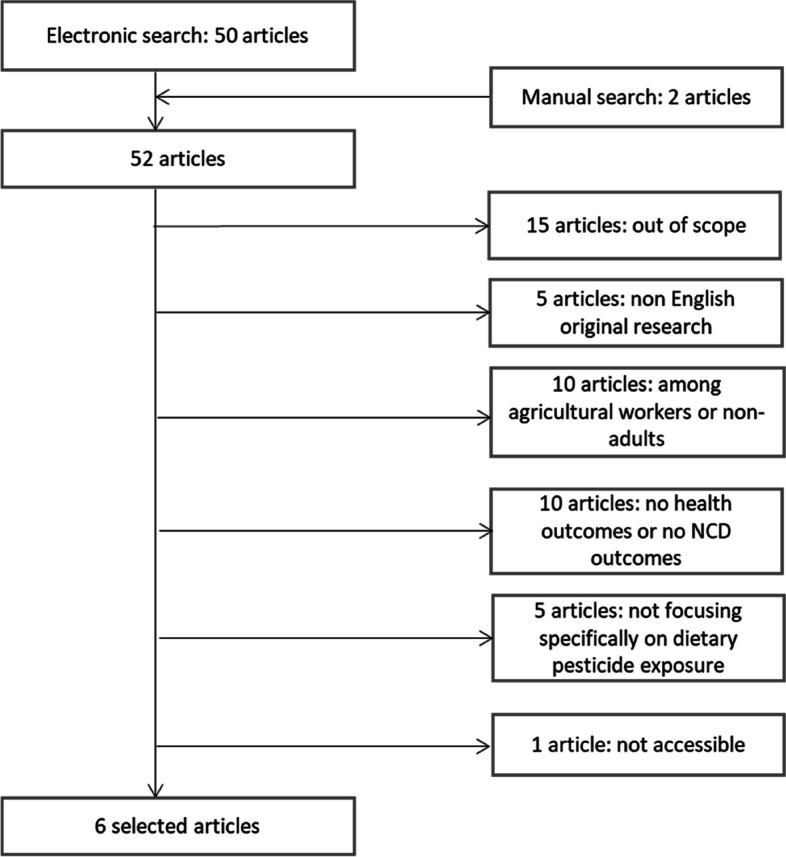
Flow chart. *NCD* Non-communicable diseases

Eligible full-text papers were independently and critically assessed by two authors (JBau, EK-G). A flowchart of the selection process is provided (Fig. 1).

### Data extraction

After study selection, the two reviewers extracted the following characteristics: the first author’s last name, year of publication, journal name, study origin, cohort name, sample size, number of cases, age at entry, sex, study duration, exposure assessment, and outcome assessment.

Then, risk estimates (hazard ratios (HR) and corresponding 95% confidence intervals (CI)), and adjustment factors were collected. In the case where several models with different adjustments were provided, the most extensively adjusted model was selected for extraction of the risk estimates.

### Risk of bias and grading of evidence

A bias analysis was conducted by JBau and EK-G (epidemiologists), according to the guidelines of NESR based on the Cochrane ROBINS-I (Risk Of Bias In Non-randomized Studies of Interventions ROBIN-I method) [15]. For each study, this analysis included the following items [15]: bias due to confounding, bias in selection of participants into the study, bias in classification of exposures, bias due to departures from intended exposures (potential change of exposure over time), bias due to missing data, bias in measurement of the outcome and bias in selection of the reported results. The different biases are graded according to four levels of judgment: low, moderate, serious, and critical, plus a grading for “no information” [15]. Agreement was observed between the two investigators for four domains (confounding, missing data, measurement of the outcomes and selection of reported results). A second evaluation was carried out for domains where disagreements were observed and a consensus was reached*.* An overall statement of bias analysis (i.e., ROB, Risk Of Bias) was provided and an overview of risk of bias for each study was illustrated using the *Robvis* tool [16]. PR, first author of two selected studies did not contribute to the decisions as regards the risk of bias analysis.

Grade of evidence was then assessed according to NESR guidelines using 5 items: the aforementioned-derived ROB, consistency, directness, precision, and generalizability, which were rated on a 4-level scale: strong, moderate, limited, or not assignable (i.e. lack of evidence or severe methodological issues). The grading terminology and definitions are provided in the publication by NESR [13].

## Results

A flowchart describing the selection of studies is shown in Fig. 1. For each excluded study, reasons for exclusion are presented in Supplemental Table 2.

Among the 52 eligible studies (of which 2 were manually identified), a total of 6 studies were selected [17–22]. The characteristics of each study are presented in Table 2.

**Table 2:** Characteristics of the included prospective studies (by decreasing year of publication)

**Authors, year and journal**	**Country**	**Design and sample**	**Follow-up and cases**	**Exposure assessment**	**Health outcomes**	**Statistical analysis and confounding factors**
Sandoval-Insausti et al., 2022 *Environ. Int*. [17]	United-States	137,378 W (NHS, 1998–2019, and NHSII, 1999–2019) and 23,502 M (HPFS, 1998–2020) free of CVD, cancer, or diabetes at baseline.	27,026 deaths, 4,318 from CVD, 6,426 from cancer, 3,081,360 person-years of follow-up.	Cumulative exposure over time of F&V (FFQ) classified as high and low-pesticide residues using the PRBS, a validated score system (contamination data from USDA PDP). F&V with PRBS ≥ 4 classified as having high-pesticide-residue status, F&V with PRBS < 4 as low-pesticide-residue status. F&V without matching PDP data as having undetermined -pesticide residue status. Two different exposures (i.e. F&V intake, considering pesticide residue status): high-pesticide and low-pesticide-residue F&V intakes.	All-cause mortality. Death recorded from state vital records, National Death Index, and reports from family members or the postal authorities. Classification of causes according to ICD-8.	Multivariable Cox model (HR, 95%CI). Confounding factors: age (time scale), BMI, race, physical activity, family history of cancer and CVD, smoking (package/y), baseline HTA, hypercholesterolemia, total energy intake, alcohol intake, modified aHEI (excluding F&V and alcohol) and hormone use for menopaused W. Intakes of high-, low- and undetermined-pesticide residue F&V were simultaneously included in all models.
Cote et al., 2022 *Am J Epidemiol* [18]	United-States	121,701 W from NHS nurses and 116,686 W from NHSII and 51,529 M from HPFS.	A total of 275 glioma cases / 2,745,862 person-years.	Cumulative exposure over time of F&V (FFQ) classified as high and low-pesticide residues using the PRBS, a validated score system (contamination data from USDA PDP). F&V with PRBS ≥ 4 classified as having high-pesticide-residue status, F&V with PRBS < 4 as low-pesticide-residue status. F&V without matching PDP data as having undetermined -pesticide residue status. Two different exposures (i.e. F&V intake, considering pesticide residue status): high-pesticide and low-pesticide-residue F&V intakes.	Self-report primary brain malignancy cases confirmed by medical record review by study investigators as diagnosis of code 191 ICD 9^th^	Multivariable Cox model (HR, 95%CI). Age as time scale stratified by calendar time. Confounding factors: age (time scale), BMI, smoking status, alcohol intake, race, physical activity, and a modified aHEI score that did not include the fruit, vegetable, and alcohol intake components, energy intake. In a supplementary model, adjustment for total flavonoid intake. Intakes of high-, low- and undetermined-pesticide residue F&V were simultaneously included in all models
Rebouillat et al., 2022 *Env Health* [19]	France	33,013 participants, mean age 53 years old, including 76% of women.	340 incident T2D cases, 169,904.28 person-years, median follow-up = 5.95 years.	Combination of baseline dietary intakes (organic/conventional consumption) with pesticide residue concentration values in plant foods. Contamination data from the European Union reference laboratory for pesticides, CVUAS. Use of WHO scenario for exposure estimation. 25 pesticides and NMF used for extraction of pesticide exposure profiles: 4 NMF-factors.	Self-report of T2D and validation using medico-administrative database, drug use and official database (CePIDC) for vital status.	Multivariable Cox model for T2D incidence (HR, 95%CI). Confounding factors: age (time scale), gender, physical activity, smoking status, educational level, occupation, household income, marital status, alcohol-free energy intake, family history of diabetes, weight, height, overall quality of the diet (measured by the PNNS-GS2 score). Additional models adjusted for provegetarian score and three scores assessing quality of foods, cDQI, pDQI and aDQI. Test for interactions between gender and sPNNS-GS2 (i.e. potential modulating factors) and NMF components.
Sandoval-Insausti et al., 2021 *Environ Int* [20]	United-States	150,830 W (NHS, 1998–2016, and NHSII, 1999–2017) and 29,486 M (HPFS, 1998–2016) without a history of cancer.	23,678 incident cancer cases, 2,862,118 person-years of follow-up. 14 years of follow-up.	Cumulative exposure over time of F&V (FFQ) classified as high and low-pesticide residues using the PRBS, a validated score system (contamination data from USDA PDP). F&V with PRBS ≥ 4 classified as having high-pesticide-residue status, F&V with PRBS < 4 as low-pesticide-residue status. F&V without matching PDP data as having undetermined -pesticide residue status. Two different exposures (i.e. F&V intake, considering pesticide residue status): high-pesticide and low-pesticide-residue F&V intakes.	Self-report of cancer confirmed by review of medical and pathology records (histology, anatomic location, and stage) by study physicians. For prostate cancer, only advanced prostate cancer was considered. Primary outcome = total cancer incidence. Selected specific sites as secondary outcomes.	Multivariable Cox model (HR, 95%CI). Confounding factors: age, height, BMI, ethnicity, physical activity, family history of cancer, physical examination in the past 2 years, history of colonoscopy or sigmoidoscopy, smoking in packyears, current multivitamin use, regular aspirin use, total energy intake, alcohol intake, and aHEI score, hormone use for menopaused W and mammography in the past 2 years in NHS and NHSII and for prostate-specific antigen testing in the past 2 years in HPFS. Intakes of high-, low- and undetermined-pesticide residue F&V were simultaneously included in all models.
Rebouillat et al., 2021 *Int J Epidemiol* [21]	France	13,149 women mean age at baseline was 60.5 years (SD = 7.39).	169 incident breast cancer cases, 57 203.70 person-years, median follow-up = 4.83 years.	Combination of baseline dietary intakes (organic/conventional consumption) with pesticide residue concentration values in plant foods. Contamination data from the European Union reference laboratory for pesticides, CVUAS. Use of WHO scenario for exposure estimation. 25 pesticides and NMF used for extraction of pesticide exposure profiles: 4 NMF-factors.	Self-report of postmenopausal breast cancer and validation using medical records and official database (CePIDC) for vital status.	Multivariable Cox for cancer incidence (HR, 95%CI). Confounding factors: age (time scale), smoking status, educational level, alcohol intake, alcohol-free energy intake, physical activity, BMI, height, family history of cancer, menopausal treatment, parity, overall quality of the diet (measured by the PNNS-GS2 score). Additional models with adjustments for residing currently in an agricultural area, the level of ultra-processed foods in the diet and the provegetarian score. Test for interactions between BMI, sPNNS-GS2 and the level of plant-based (i.e. modulating factors) and NMF components.
Chiu et al., 2019 *Environ Int* [22]	United-States	145,789 W and 24,353 M free of CVD and cancer from the NHS (NHS: 1998–2012), the NHS-II (1999–2013), and the HPFS (HPFS: 1998–2012).	3,707 incident CHD cases, 2,241,977 person-years.	Cumulative exposure over time of F&V (FFQ) classified as high and low-pesticide residues using the PRBS, a validated score system (contamination data from USDA PDP). F&V with PRBS ≥ 4 classified as having high-pesticide-residue status, F&V with PRBS < 4 as low-pesticide-residue status. F&Vs without matching PDP data as having undetermined -pesticide residue status. Two different exposures (i.e. F&V intake, considering pesticide residue status): high-pesticide and low-pesticide-residue F&V intakes.	Validation of cases based on medical records or telephone interviews. Non-fatal myocardial infarction confirmed using WHO criteria on the basis of symptoms plus elevated specific cardiac enzymes or electrocardiogram changes indicative of new ischemia.	Multivariable Cox model (HR, 95%CI). Confounding factors: age (time scale), BMI, race, physical activity, family history of myocardial infarction/diabetes, smoking status, baseline HTA, hypercholesterolemia, and diabetes, current multivitamin use, current aspirin use, total energy intake, alcohol intake, aHEI (excluding F&V and alcohol), hormone use for menopaused W (in NHS and NHSII), oral contraceptive (in NHSII). Intakes of high-, low- and undetermined-pesticide residue F&V were simultaneously included in all models.

*Abbreviations*: *95%CI* 95%Confidence Intervals, *aDQI* animal-based Diet Quality Index, *aHEI* Alternate Healthy Eating Index score, *cDQI* Comprehensive Diet Quality Index, *CHD* Coronary heart diseases, *CVD* Cardiovascular diseases, *CVUAS* Chemisches und Veterinäruntersuchungsamt Stuttgart, *FFQ* Food Frequency Questionnaire, *F&V* fruits and vegetables, *HPFS* Health Professional Follow-up Study, *HR* hazard ratio, *HTA* hypertension, *ICD* International Classification of diseases, *M* men, *NHS* Nurses’ Health Study, *PDP* Pesticide Data Program, *pDQI* plant-based Diet Quality Index, *PRBS* Pesticide Residue Burden Score, *sPNNS-GS2* simplified *Programme National Nutrition Santé*-guidelines score 2, *T2D* type 2 diabetes, *USDA *United States Department of Agriculture,* W* women, *WHO* World health organization

### Method for assessment of pesticide exposure

#### The Pesticide Residue Burden Score (PRBS)

Pesticide exposure was assessed in the US cohort studies (conducted in the Nurses’ Health Study (NHS, NHSII) and Health Professional Follow-up Study (HPFS) [17, 18, 20, 22]), using a broad indicator, named PRBS. This indicator combines pesticide surveillance data from the USDA (> 400 different pesticide residues) and food frequency questionnaire (FFQ) consumption data. It allows to classify individuals according to their pesticide residue exposure from fruits and vegetable (F&V) intake.

The PRBS has been validated against 1) urinary concentrations of pesticide biomarkers of organophosphates and pyrethroids, and the 2,4-dichlorophenoxyacetic acid, measured in 90 men participating in the Environment and Reproductive Health cohort study [23] and 2) pesticide metabolites, assessed in 3,679 individuals of the US nationally representative survey NHANES [24].

This index is based on the three following criteria (from the pesticide surveillance program): the percentage of F&V samples with any detectable pesticide residues, the percentage of F&V samples with any pesticide residues above tolerance levels, and the percentage of F&V samples with three or more individual detectable pesticides. For each contamination index, FFQ-F&V are ranked according to their tertile-distribution (assigning 0, 1 or 2 for each respective tertile). Each score is then summed up to obtain the PRBS, which ranges from 0 to 6. A high-pesticide-residue status is given to F&V with a score ≥ 4, a low-pesticide-residue status is given to F&V with a score lower than 4, and an undetermined-pesticide-residue status is given to F&V without contamination data. Cumulative average intakes (per servings per day) of high-, low- and undetermined-pesticide residue F&V are then calculated for each participant and modeled as continuous variables and as quintiles.

#### Plant-based food exposure profiles derived from contamination data

In the French studies [19, 21], pesticide exposure was estimated by combining plant-based food consumption and CVUAS contamination data for 25 specific pesticides, including substances authorized in organic farming. Exposure profiles were then identified using the non-negative matrix factorization method [25]. This method is a non-supervised dimensionality reduction technique, developed by Lee et Seung [25], especially adapted for parsimonious data subject to the constraints of assay methods (limits of detection and quantification).

### Risk of bias assessment

Studies were all conducted in the United States or France, and involved from 13,149 [21] to 180,316 participants [20]. The follow-up duration varied from 5 [21] to 20y [17]. Investigated outcomes included mortality (overall and by causes), cancer (notably breast cancer and glioma), coronary heart diseases (CHD), and type 2 diabetes (T2D).

**Fig. 2:**
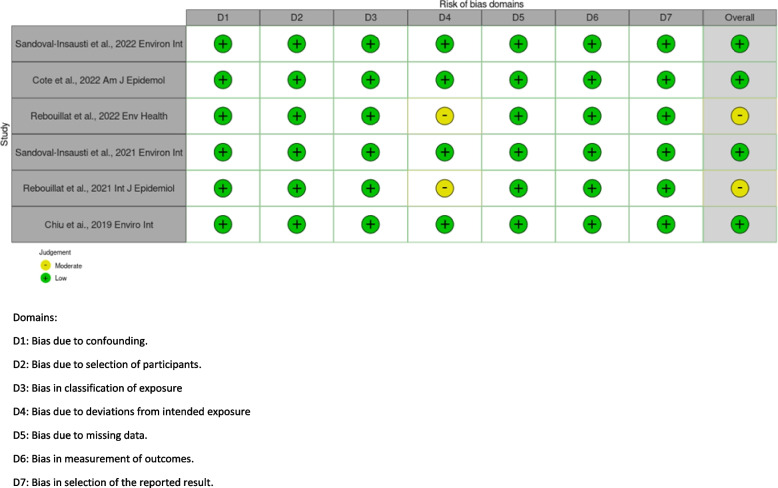
Bias analysis for each study

A risk of bias analysis for each study was conducted and is detailed in Fig. 2. Overall, the quality of reviewed studies was high, studies were rated as having low or moderate risk of bias.

### Association study results

The main findings are reported in Table 3.

**Table 3:** Conclusion statements from included studies (by decreasing year of publication)

**Authors, year, journal**	**Main findings**
Sandoval-Insausti et al., 2022 *Environ Int* [17]	Consumptions of ≥ 4 servings/day of low-pesticide-residue F&V were linked to 36% lower risk of total **mortality** relative to consuming < 1 serving/day (total mortality, low pesticides F&V: HR_Q5vsQ1_ = 0.64, 95%CI (0.59, 0.68)). Conversely, intake of high-pesticide-residue F&V was unrelated to mortality (corresponding estimate for high-pesticide residue F&V intake was 0.93 (95% CI: 0.81, 1.07)). Pattern similar across the three most frequent causes of death (cardiovascular diseases, cancer and respiratory diseases).
Cote et al., 2022 *Am J Epidemiol* [18]	An association was observed between intake of high-pesticide-residue F&V and **glioma** in NHS (multivariable-adjusted HR = 2.99, 95%CI: 1.38, 6.44 comparing highest with lowest quintile, P for trend = 0.02). No association identified in NHSII (multivariable-adjusted HR = 0.52, 95%CI: 0.19, 1.45, P for trend = 0.20) or HPFS (multivariable-adjusted HR = 1.01, 95%CI: 0.42, 2.45, P for trend = 0.39). No significant associations were detected with low-pesticide-residue F&V consumption or overall consumption in any cohort.
Rebouillat et al., 2022 *Env Health* [19]	Positive association between NMF component 1 (reflecting highest exposure to several synthetic pesticides) and **T2D** risk on the whole sample: HR_Q5vsQ1_ = 1.47, 95%CI (1.00, 2.18). NMF Component 3 (reflecting low exposure to several synthetic pesticides) was associated with a decrease in T2D risk, among those with high dietary quality only (high adherence to French dietary guidelines, including high plant foods consumption): HR_Q5vsQ1_ = 0.31, 95%CI (0.10, 0.94). NMF Component 2 associated with a higher T2D risk in the 3rd tertile of sPNNS-GS2 score (high adherence to French dietary guidelines): P for trend = 0.03.
Sandoval-Insausti et al., 2021 *Environ Int* [20]	The HR (95%CI) of **cancer** per 1 serving/day increase in intake were 0.99, 95%CI (0.97, 1.01) for high- and 1.01, 95%CI (0.99, 1.02) for low-pesticide-residue F&V intake. No association between high pesticide-residue F&V intake ([HR, 95%CI comparing Q5 vs Q1 of intake] and risk of specific sites, including malignancies previously linked to occupational pesticide exposure or organic foods: lung [1.17 95%CI (0.95, 1.43)], non-Hodgkin lymphoma [0.89 95%CI (0.72, 1.09)], prostate [1.31 95%CI (0.88, 1.93)]) or breast [1.03 95%CI (0.94, 1.31)]).
Rebouillat et al., 2021 *Int J Epidemiol* [21]	Negative associations between Component 3, reflecting low exposure to synthetic pesticides, and postmenopausal **BC** risk: HR_Q5vsQ1_ = 0.57; 95%CI (0.34;0.93), P for trend = 0.006. Positive association between Component 1 score (highly correlated to chlorpyrifos, imazalil, malathion, thiabendazole) and post-menopausal BC risk was found specifically among women with overweight or obesity, HR_Q5vsQ1_ = 4.13; 95%CI (1.50;11.44), P for trend = 0.006. No associations were detected for the other components.
Chiu et al., 2019 *Environ Int* [22]	Pooled: High-pesticide-residue F&V: multivariable-adjusted for **CHD**: HR_Q5vsQ1_ = 1.06, 95%CI (0.92, 1.21), P for trend = 0.45. Low-pesticide-residue F&V: HR_Q5vsQ1_ = 0.82, 95%CI (0.71, 0.94), P for trend = 0.001.

*Abbreviations*: *95%CI* 95%Confidence Intervals, *BC* breast cancer, *CI* confidence interval, *CHD* Coronary heart diseases, *F&V* fruits and vegetables, *HPFS* Health Professional Follow-up Study, *HR* hazard ratio, *NHS* Nurses’ Health Study, *NMF* non-negative matrix factorization, *Q* quartile or quintile, *T2D* type 2 diabetes

#### Cancer

Three studies investigating the risk of cancer in relation to dietary pesticide exposure were identified. One study conducted in the three cohorts of American men and women reported no association between pesticide residue levels by F&V status and risk of cancer, regardless of the location [20]. Another study, conducted within the same cohorts, investigating the risk of glioma reported a higher risk among NHS participants with high intake of high-residue F&V (HR_Q5 vs. Q1_ = 2.99, 95%CI (1.38; 6.44)) but no association was observed in the other cohorts. In addition, no associations were observed when studying overall or low-residue F&V consumption [18]. The study conducted among French women from the NutriNet-Santé study reported a lower risk of postmenopausal breast cancer among women highly exposed to a mixture of pesticides authorized in organic farming (negatively correlated to synthetic pesticides) (HR_Q5 vs. Q1_ = 0.57; 95%CI (0.34;0.93)) [21]. A positive association between postmenopausal breast cancer and the mixture correlated to chlorpyrifos, imazalil, malathion, thiabendazole was also detected among women with overweight (including obesity): HR_Q5 vs. Q1_ = 4.13; 95%CI (1.50;11.44). Other NMF-extracted components were not associated with the risk of postmenopausal breast cancer.

#### Heart and metabolic diseases

A single study conducted in the same American cohorts evaluated the association between high and low-pesticide residue F&V and CHD [22]. A negative association between intake of low-pesticide residue F&V and the risk of CHD, was observed: HR _Q5 vs. Q1_ = 0.82; 95%CI (0.71, 0.94). On the opposite, no association was detected for intake of high-pesticide residue F&V.

An association between pesticide exposure and the risk of T2D was found in the NutriNet-Santé study, with a higher risk among participants with higher exposure to a profile of synthetic pesticides: HR_Q5_ _vs. Q1_ = 1.47, 95%CI (1.00, 2.18) [19]. A pesticide exposure profile weakly correlated to synthetic pesticides and highly correlated with pesticides authorized in organic farming, was associated with a lower risk of T2D: HR_Q5_ _vs. Q1_ = 0.31, 95%CI (0.10, 0.94), only among individuals with overall high dietary quality.

#### Mortality

Based on data from the three very large US prospective cohorts, another study examined the association of intake of F&V according to pesticide residue status with mortality and found an inverse association with intake of low-pesticide-residue F&V: HR_Q5vsQ1_ 0.64, 95%CI (0.59, 0.68), but not with intake of high-pesticide-residue F&V [17]. This trend was observed for mortality caused by CVD, cancer and respiratory diseases.

### Quality of evidence grading

Grading of evidence for each health outcome is presented in Table 4. With regard to cancer, 3 studies were available (with one reporting no association and two a positive association), the level of evidence therefore can be considered as low. For the other outcomes, only one was available, leading to a non-assignable grade for the association.

**Table 4:** Grade of evidence for each outcome (by decreasing year of publication)^a^

**Auteur, date, nom du journal**	**Risk of bias**	**Consistency**	**Directness**	**Precision**	**Generalizability**
**Mortality**	**Low**	**Not attributable**	**Strong**	**Strong**	**Strong**
*Sandoval-Insausti *et al*., 2022* [17]	*Low*		*Strong*	*Strong*	*Strong*
**Cancer**	**Moderate**	**Low**	**Strong**	**Strong**	**Strong**
*Cote *et al*., 2022* [18]	*Low*		*Strong*	*Strong*	*Strong*
*Rebouillat *et al*., 2021* [21]	*Moderate*		*Strong*	*Strong*	*Strong*
*Sandoval-Insausti *et al*., 2021* [20]	*Low*		*Strong*	*Strong*	*Strong*
**Cardiovascular diseases**	**Low**	**Not attributable**	**Strong**	**Strong**	**Strong**
*Chiu et al., 2019* [22]	*Low*		*Strong*	*Strong*	*Strong*
**Diabetes**	**Moderate**	**Not attributable**	**Strong**	**Strong**	**Strong**
*Rebouillat *et al*., 2022* [19]	*Moderate*		*Strong*	*Strong*	*Strong*

^a^Not attributable: the consistency cannot be evaluated when only one study is included

## Discussion

### Quality of the included studies

Studies were based on large samples, validated outcomes limiting misclassification, and exposure was derived from detailed food consumption and contamination data. Included studies also accounted for a wide range of potential confounding factors, including dietary patterns which are strongly correlated to pesticide exposure [26, 27]. However, residual confounding can never be excluded from observational studies, even when a wide range of confounders are considered. The studies conducted in the American health cohorts used an index previously validated aiming to classify F&V according to pesticide contamination level. Of note, F&V were classified into simple pesticide residue status categories, which meant that actual quantitative contamination information was not considered when estimating pesticide exposure while the contamination range can be large within both low and high contaminated F&V categories. In particular, the six validated categories of the PRBS were collapsed in binary variables (high/low) in these studies.

In these studies, farming practices with different regulations for pesticide use (i.e. organically or conventionally-grown) were not distinguished nor intake of other potentially pesticide-contaminated foods such as cereals. In contrast, the French studies [19, 21] accounted for detection/quantification limits to build pessimist and optimistic exposure scenario, as recommended by the WHO [28].

However, in the French studies, follow-up duration was short (median follow-up time was approximately 5 years) while it was much longer in the American studies, leading to a very high statistical power in the US studies. In addition, all studies were conducted in Western populations and therefore generalizability is limited for low- or middle-income countries. The American studies focused on F&V while the French studies focused on all foods from plant origin, limiting comparison.

We did not identify any RCT. The level of evidence from prospective observational studies is not as high as that from RCT, however pesticide exposure is a typical case where RCTs are hardly feasible due to technical and ethical issues [29].

### Grading level of evidence

Overall, the studies reporting an association between pesticide exposure and NCD outcomes pointed in the direction of a deleterious effect. The study on mortality risk in the general population showed no association with consumption of high-pesticide-residue F&V, as opposed to consumption of low-pesticide-residue F&V which were linked to a protective effect. Among the three studies modeling cancer incidence, two reported a positive association between higher dietary exposure to pesticide residues and risk of glioma and postmenopausal breast cancer (specifically among women with overweight or obesity), respectively and the third study reported no association with all studied sites. The French study focusing on T2D risk showed an association with high exposure to a specific profile of synthetic pesticides. A lower risk of CHD was observed among high American consumers of low-pesticide-residue F&V, while no protective effects were detected with the consumption of high-pesticide-residue F&V. Overall, the number of studies per outcome was very limited, limiting the possibility to grade the level of evidence. For other outcomes than cancer, the grading was not assignable as only one was available. Overall – although not many – studies were of high quality and suggested a role of pesticide exposure through food on health, in particular, risk of cancer and subsequent mortality. This seems consistent with a recent systematic review on pesticides and risk of cancer [11].

### Mechanistic pathways

The biological mechanisms through which pesticides can alter biological functioning have been extensively described in experimental studies. Pesticides can affect human health through multiple pathways involving several target organs. In turn, these alterations result in a higher risk of various pathologies (cancers, CVD, respiratory pathologies, neurodegenerative diseases, etc.) [30]. First of all, some pesticides (e.g. organophosphorus compounds) can induce dysregulations of carbohydrate and lipid metabolisms [31], through several underlying mechanisms involving oxidative stress, alterations of insulin secretion, paraoxonase inhibition, or cholinesterase inhibition [32]. Some contaminants may also influence adipocyte proliferation and differentiation by interacting with different nuclear receptors [33, 34]. Next, pesticides may cause genetic alterations (mutation and premutagenic alterations) by direct interactions with the genetic material, leading to DNA damage or chromosomal aberrations [30]. Pesticides may also induce epigenetic modifications such as DNA methylation [30]. Third, pesticides may act as endocrine disruptors, i.e. they can interfere with the synthesis, secretion, transport, binding, action, metabolism or elimination of hormones [35, 36]. The endocrine disrupting properties of pesticides (e.g. organophosphorus moieties) have been indeed widely documented [37] and refer to their ability to mimic estrogenic function by acting as a ligand for receptors, converting other steroids to active estrogens, or increasing the expression of estrogen-sensitive genes [38]. In addition, anti-androgenic effects, through inhibition of androgen-binding receptors, have also been described for organochlorine insecticides, carbamates, and triazines. Finally, organophosphates may inhibit thyroid hormone receptors and pyrethroids may inhibit the action of progesterone [38]. Although not clearly documented, the gut microbiota could potentially play a role in the relationship between pesticides and health notably by inducing a reduction in prevalence of *Bifidobacteria* and *Lactobacillus* and an increase in *Enterococcus* and *Bacteroides* [39].

### Recommendations for further work

Development of well-conducted *ad hoc* studies is warranted to increase the level of evidence on the impact of dietary pesticide exposure on diet-related NCD. Surveillance components have been included in monitoring surveys in some countries, but long-term prospective population-based studies aiming at estimating the association between dietary exposure to pesticides and human health are needed. Although of interest, accurate exposure measurement (e.g. using biomarkers) is currently expensive and requires high logistic resources limiting its use in large-scale cohorts. Moreover, urinary biomarkers do not generally allow to consider long-term exposure and usually reflect overall exposure, making it difficult to disentangle the various routes of exposure (dietary, respiratory, and cutaneous). This does not make it possible to focus specifically on the dietary route, which is the main route of exposure in the general population, and for which preventive individual and collective actions can be undertaken. To overcome these aspects, the matching of consumption and contamination data is a relevant option which requires: 1) the existence of high-quality consumption and contamination databases, covering a large panel of regions including countries from global South, and a long time period, 2) easy access to these databases and 3) interoperability of the consumption and contamination databases. In addition, a major issue concerns the comparison between studies. For example, regulations vary across countries and change over time. The way pesticide exposure is assessed can also considerably differ depending on the study (e.g. single molecules or mixture of molecules can be considered). Different types of variables (e.g. exposure vs non-exposure or low-level vs high-level contaminated foods) can also be used to reflect different exposure levels. To our knowledge, there are indeed no official international recommendations providing standardized guidance on the way to estimate dietary exposure, including the type of molecules to be prioritized, and the type of statistical modelling to be conducted. There is, therefore, an urgent need to develop a new generation of epidemiological studies considering a common methodological framework to assess exposure to pesticides through diet, in order to better characterize the risk for individuals, and to conduct meta-analyses in the future. While working on specific residues allows comparison across studies, it is also of great importance to focus on mixture rather than single molecules, for the same reasons dietary patterns rather than single nutrients are studied in nutritional epidemiology [40, 41]. It will allow to consider potential synergic, antagonist, or cumulative effects. Exposure is the result of each food’s contamination but also depends on consumption patterns. When studying exposure to pesticides in the general population, diet is the main route (chronic exposure to low doses), thus the regional context and current regulation should be considered to focus on authorized active substances, as an important public health target. This implies that updated analyses are conducted, to account for changes in exposure patterns following newly banned active substances. Then, farming practices for food production are strong determinants and should be better assessed.

## Conclusion

Finally, studies investigating the impact of exposure to dietary pesticides on the onset and progression of NCD in adults are scarce. In addition, studies were conducted in the Western context (France and the United States) and studies in other settings are necessary. Furthermore, prospective studies using detailed pesticide exposure with various endpoints are warranted and various sources of pesticide exposure should also be controlled for. In terms of public health implications, a reduction and minimization of pesticide exposure, notably by the dietary route, may be an important lever for health promotion at the population level. The most harmful residues should be subject to increased monitoring to determine priorities for prohibition and research to propose alternatives.

### Supplementary Information

**Additional file 1: Supplemental Method 1.** Request for research. **Supplemental Table 1.** PRISMA Checklist. **Supplemental Table 2.** Excluded studies and reasons for exclusion.

## Data Availability

Researchers from public institutions can submit a collaboration request including information on the institution and a brief description of the project to collaboration@etude-nutrinet-sante.fr. All requests will be reviewed by the steering committee of the NutriNet-Sante study. A financial contribution may be requested. If the collaboration is accepted, a data access agreement will be necessary and appropriate authorizations from the competent administrative authorities may be needed. In accordance with existing regulations, no personal data will be accessible.

## Abbreviations

aDQI: Animal-based Diet Quality Index
CVUAS: Chemisches und Veterinäruntersuchungsamt Stuttgart
cDQI: Comprehensive Diet Quality Index
CHD: Coronary heart diseases
CVD: Cardiovascular diseases
FFQ: Food frequency questionnaire
F&V: Fruits and vegetables
HPFS: Health Professional Follow-up Study
ICD: International Classification of Diseases
NESR: Nutrition Evidence Systematic Review
NCD: Non-communicable diseases
NHS: Nurses’ Health Study
NHSII: Nurses’ Health Study II
ROB: Risk Of Bias
ROBINS-I: Risk Of Bias In Non-randomized Studies of Interventions
pDQI: Plant-based Diet Quality Index
PRBS: Pesticide Residue Burden Score
T2D: Type 2 Diabetes
USDA: United States Department of Agriculture
WHO: World Health Organization
