# Supplementary material for: Dietary pesticide exposure and non-communicable diseases and mortality: a systematic review of prospective studies among adults
Source: Environ Health. 2023 Oct 31;22:76. doi: 10.1186/s12940-023-01020-8 (PMC10617043; doi:10.1186/s12940-023-01020-8)
Supplement: Supplementary file 1 — Additional file 1: Supplemental Method 1. Request for research. Supplemental Table 1. PRISMA Checklist. Supplemental Table 2. Excluded studies and reasons for exclusion. [file 12940_2023_1020_MOESM1_ESM.docx]

**Dietary pesticide exposure and non-communicable diseases and mortality: a systematic review of prospective studies among adults**

Table of contents

[Supplemental Method 1: Request for research 2](#_Toc146118324)

[Supplemental Table 1: PRISMA Checklist 3](#_Toc146118325)

[Supplemental Table 2: Excluded studies and reasons for exclusion 5](#_Toc146118326)

# Supplemental Method 1: Request for research

(pesticide* **OR** glyphos* **OR** pyrethr* **OR** fungicid* **OR** insecticid* **OR** herbicid* **OR** residues* **OR** organophos* **OR** carbamate* )[TITLE/ABSTRACT]

**AND** (dietary exposure or food exposure)

**AND** prospective cohort

**NOT** (prenat*)

# Supplemental Table 1: PRISMA Checklist

| **Section and Topic** | **Item #** | **Checklist item** | **Location where item is reported** |
| --- | --- | --- | --- |
| **TITLE** |  |  |  |
| Title | 1 | Identify the report as a systematic review. | P1 |
| **ABSTRACT** |  |  |  |
| Abstract | 2 | See the PRISMA 2020 for Abstracts checklist. |  |
| **INTRODUCTION** |  |  |  |
| Rationale | 3 | Describe the rationale for the review in the context of existing knowledge. | P4-5 |
| Objectives | 4 | Provide an explicit statement of the objective(s) or question(s) the review addresses. | P5 |
| **METHODS** |  |  |  |
| Eligibility criteria | 5 | Specify the inclusion and exclusion criteria for the review and how studies were grouped for the syntheses. | P5-6 |
| Information sources | 6 | Specify all databases, registers, websites, organisations, reference lists and other sources searched or consulted to identify studies. Specify the date when each source was last searched or consulted. | P5-6 |
| Search strategy | 7 | Present the full search strategies for all databases, registers and websites, including any filters and limits used. | P5-6 |
| Selection process | 8 | Specify the methods used to decide whether a study met the inclusion criteria of the review, including how many reviewers screened each record and each report retrieved, whether they worked independently, and if applicable, details of automation tools used in the process. | P6 |
| Data collection process | 9 | Specify the methods used to collect data from reports, including how many reviewers collected data from each report, whether they worked independently, any processes for obtaining or confirming data from study investigators, and if applicable, details of automation tools used in the process. | P6-7 |
| Data items | 10a | List and define all outcomes for which data were sought. Specify whether all results that were compatible with each outcome domain in each study were sought (e.g. for all measures, time points, analyses), and if not, the methods used to decide which results to collect. | P6-7, P21-26 |
|  | 10b | List and define all other variables for which data were sought (e.g. participant and intervention characteristics, funding sources). Describe any assumptions made about any missing or unclear information. | P6-7, P21-26 |
| Study risk of bias assessment | 11 | Specify the methods used to assess risk of bias in the included studies, including details of the tool(s) used, how many reviewers assessed each study and whether they worked independently, and if applicable, details of automation tools used in the process. | P6-7 |
| Effect measures | 12 | Specify for each outcome the effect measure(s) (e.g. risk ratio, mean difference) used in the synthesis or presentation of results. | P6 |
| Synthesis methods | 13a | Describe the processes used to decide which studies were eligible for each synthesis (e.g. tabulating the study intervention characteristics and comparing against the planned groups for each synthesis (item #5)). | P6-7 |
|  | 13b | Describe any methods required to prepare the data for presentation or synthesis, such as handling of missing summary statistics, or data conversions. | - |
|  | 13c | Describe any methods used to tabulate or visually display results of individual studies and syntheses. | P8, P33 |
|  | 13d | Describe any methods used to synthesize results and provide a rationale for the choice(s). If meta-analysis was performed, describe the model(s), method(s) to identify the presence and extent of statistical heterogeneity, and software package(s) used. | P7, P31 |
|  | 13e | Describe any methods used to explore possible causes of heterogeneity among study results (e.g. subgroup analysis, meta-regression). | - |
|  | 13f | Describe any sensitivity analyses conducted to assess robustness of the synthesized results. | - |
| Reporting bias assessment | 14 | Describe any methods used to assess risk of bias due to missing results in a synthesis (arising from reporting biases). | P7, P31 |
| Certainty assessment | 15 | Describe any methods used to assess certainty (or confidence) in the body of evidence for an outcome. | P7, P31 |
| **RESULTS** |  |  |  |
| Study selection | 16a | Describe the results of the search and selection process, from the number of records identified in the search to the number of studies included in the review, ideally using a flow diagram. | P8-9 |
|  | 16b | Cite studies that might appear to meet the inclusion criteria, but which were excluded, and explain why they were excluded. | P6, supplementary material |
| Study characteristics | 17 | Cite each included study and present its characteristics. | P7-9, P22-26 |
| Risk of bias in studies | 18 | Present assessments of risk of bias for each included study. | P29 |
| Results of individual studies | 19 | For all outcomes, present, for each study: (a) summary statistics for each group (where appropriate) and (b) an effect estimate and its precision (e.g. confidence/credible interval), ideally using structured tables or plots. | P10-12, P24-27 |
| Results of syntheses | 20a | For each synthesis, briefly summarise the characteristics and risk of bias among contributing studies. | P22-26, P31 |
|  | 20b | Present results of all statistical syntheses conducted. If meta-analysis was done, present for each the summary estimate and its precision (e.g. confidence/credible interval) and measures of statistical heterogeneity. If comparing groups, describe the direction of the effect. | P9-11, P27-28 |
|  | 20c | Present results of all investigations of possible causes of heterogeneity among study results. | - |
|  | 20d | Present results of all sensitivity analyses conducted to assess the robustness of the synthesized results. | - |
| Reporting biases | 21 | Present assessments of risk of bias due to missing results (arising from reporting biases) for each synthesis assessed. | P31 |
| Certainty of evidence | 22 | Present assessments of certainty (or confidence) in the body of evidence for each outcome assessed. | P10-11, P29 |
| **DISCUSSION** |  |  |  |
| Discussion | 23a | Provide a general interpretation of the results in the context of other evidence. | P11-15 |
|  | 23b | Discuss any limitations of the evidence included in the review. | P11-15 |
|  | 23c | Discuss any limitations of the review processes used. | P11-15 |
|  | 23d | Discuss implications of the results for practice, policy, and future research. | P14-15 |
| **OTHER INFORMATION** |  |  |  |
| Registration and protocol | 24a | Provide registration information for the review, including register name and registration number, or state that the review was not registered. | P5 |
|  | 24b | Indicate where the review protocol can be accessed, or state that a protocol was not prepared. | P5 |
|  | 24c | Describe and explain any amendments to information provided at registration or in the protocol. | P5 |
| Support | 25 | Describe sources of financial or non-financial support for the review, and the role of the funders or sponsors in the review. | P1, P17 |
| Competing interests | 26 | Declare any competing interests of review authors. | P17 |
| Availability of data, code and other materials | 27 | Report which of the following are publicly available and where they can be found: template data collection forms; data extracted from included studies; data used for all analyses; analytic code; any other materials used in the review. | P16 |

# Supplemental Table 2: Excluded studies and reasons for exclusion

|  | Authors | Year of publication | Title | Reasons for exclusion |
| --- | --- | --- | --- | --- |
| 1 | Sadeghi H et al. | 2023 | Dietary omega-6/omega-3 fatty acids and risk of prostate cancer; Is there any potential interaction by organophosphate insecticides among the agricultural health study population | Out of scope |
| 2 | Bliznashka L et al. | 2023 | Pregnancy pesticide exposure and child development in low- and middle-income countries: A prospective analysis of a birth cohort in rural Bangladesh and meta-analysis | Infants |
| 3 | Melnik BC et al. | 2023 | The Role of Cow's Milk Consumption in Breast Cancer Initiation and Progression | Out of scope |
| 4 | David CP et al. | 2023 | Fruit and vegetable consumption, pesticide residue intake from consumption of fruits and vegetables, and risk of uterine fibroids | Fertility |
| 5 | Karavasiloglou N et al. | 2022 | Sustainable Diets and Cancer: a Systematic Review | Review |
| 6 | Garzia NA et al. | 2022 | Pesticide residue intake from fruit and vegetable consumption and risk of laparoscopically confirmed endometriosis | Non-accessible |
| 7 | Knapke ET et al. | 2022 | Environmental and occupational pesticide exposure and human sperm parameters: A Navigation Guide review | Review |
| 8 | Schildroth S et al. | 2021 | Correlates of Persistent Endocrine-Disrupting Chemical Mixtures among Reproductive-Aged Black Women | No health outcomes |
| 9 | Desalegn AA et al. | 2021 | A case-cohort study of perinatal exposure to potential endocrine disrupters and the risk of cryptorchidism in the Norwegian HUMIS study | Infants |
| 10 | Mancini FR et al. | 2021 | Identification of chemical mixtures to which women are exposed through the diet: Results from the French E3N cohort | No health outcomes |
| 11 | Kesse-Guyot E et al. | 2020 | [Prospective association between organic food consumption and the risk of type 2 diabetes: findings from the NutriNet-Santé cohort study](https://pubmed.ncbi.nlm.nih.gov/33167995/) | Not dietary pesticide exposure |
| 12 | Gilden R et al. | 2020 | Gestational Pesticide Exposure and Child Respiratory Health | Children |
| 13 | Abou Ghayda R et al. | 2020 | Peripubertal serum concentrations of organochlorine pesticides and semen parameters in Russian young men | Adolescents |
| 14 | Wesselink AK et al. | 2020 | Pesticide residue intake from fruits and vegetables and fecundability in a North American preconception cohort study. | Fertility |
| 15 | Orta OR et al. | 2020 | [Correlates of organochlorine pesticide plasma concentrations among reproductive-aged black women](https://pubmed.ncbi.nlm.nih.gov/32182481/) | No health outcomes |
| 16 | El-Zaemey S et al. | 2019 | [Animal farming and the risk of lymphohaematopoietic cancers: a meta-analysis of three cohort studies within the AGRICOH consortium](https://pubmed.ncbi.nlm.nih.gov/31302607/) | Out of scope |
| 17 | Messerlian C et al. | 2018 | Organophosphate flame-retardant metabolite concentrations and pregnancy loss among women conceiving with assisted reproductive technology | Out of scope |
| 18 | Chiu YH et al. | 2018 | Maternal intake of pesticide residues from fruits and vegetables in relation to fetal growth | Fetus |
| 19 | Carignan CC et al. | 2018 | Paternal urinary concentrations of organophosphate flame retardant metabolites, fertility measures, and pregnancy outcomes among couples undergoing in vitro fertilization | Out of scope |
| 20 | Timmermann CAG et al. | 2017 | [Secondary sex ratio in relation to exposures to polychlorinated biphenyls, dichlorodiphenyl dichloroethylene and methylmercury](https://pubmed.ncbi.nlm.nih.gov/29195489/) | Out of scope |
| 21 | Chiu YH et al. | 2018 | Association Between Pesticide Residue Intake From Consumption of Fruits and Vegetables and Pregnancy Outcomes Among Women Undergoing Infertility Treatment With Assisted Reproductive Technology | Fertility |
| 22 | Miguel V et al. | 2018 | The Role of MicroRNAs in Environmental Risk Factors, Noise-Induced Hearing Loss, and Mental Stress | Out of scope |
| 23 | Callahan CL et al. | 2017 | Consumption of Lake Ontario sport fish and the incidence of colorectal cancer in the New York State Angler Cohort Study (NYSACS) | Out of scope |
| 24 | Boulanger M et al. | 2017 | [Agricultural exposure and risk of bladder cancer in the AGRIculture and CANcer cohort](https://pubmed.ncbi.nlm.nih.gov/27815726/) | Agricultural workers |
| 25 | Tagiyeva N et al. | 2016 | Occupational exposure to asthmagens and adult onset wheeze and lung function in people who did not have childhood wheeze: A 50-year cohort study. | Not dietary pesticide exposure |
| 26 | Yang XL et al. | 2015 | Related factors and prevalence of Parkinson's disease among Uygur residents in Hetian, Xinjiang Uygur Autonomous Region | Not dietary pesticide exposure |
| 27 | Landgren O et al. | 2015 | Agent Orange Exposure and Monoclonal Gammopathy of Undetermined Significance: An Operation Ranch Hand Veteran Cohort Study | Out of scope |
| 28 | Hu R et al. | 2015 | Long- and short-term health effects of pesticide exposure: a cohort study from China | Agricultural workers |
| 29 | Chiu YH et al. | 2015 | Fruit and vegetable intake and their pesticide residues in relation to semen quality among men from a fertility clinic | Fertility |
| 30 | Torjusen H et al. | 2014 | Reduced risk of pre-eclampsia with organic vegetable consumption: results from the prospective Norwegian Mother and Child Cohort Study | Not dietary pesticide exposure |
| 31 | Zhang J et al. | 2013 | Exposure to pyrethroids insecticides and serum levels of thyroid-related measures in pregnant women | Not non-communicable diseases |
| 32 | Wesseling et al. | 2013 | Resolving the enigma of the mesoamerican nephropathy: a research workshop summary | Out of scope |
| 33 | Lam T et al. | 2013 | Predictors of serum chlorinated pesticide concentrations among prepubertal Russian boys | Children |
| 34 | Tual S et al. | 2013 | Agricultural exposures and chronic bronchitis: findings from the AGRICAN (AGRIculture and CANcer) cohort | Agricultural workers |
| 35 | Bräuner EV et al. | 2012 | Predictors of adipose tissue concentrations of organochlorine pesticides in a general Danish population | Not non-communicable diseases |
| 36 | Guldner L et al. | 2010 | Pesticide exposure of pregnant women in Guadeloupe: ability of a food frequency questionnaire to estimate blood concentration of chlordecone | Comparative study |
| 37 | Nakai K et al. | 2009 | [Tohoku study of child development and exposure assessment] | Article in japanese |
| 38 | Mendez MA et al. | 2009 | Seafood consumption in pregnancy and infant size at birth: results from a prospective Spanish cohort | Out of scope |
| 39 | Jakszyn P et al. | 2009 | Serum levels of organochlorine pesticides in healthy adults from five regions of Spain | No health outcomes |
| 40 | Fleshner N et al. | 2004 | [Dietary fat and prostate cancer](https://pubmed.ncbi.nlm.nih.gov/14713748/) | Out of scope |
| 41 | Shipp AM et al. | 2000 | Determination of a site-specific reference dose for methylmercury for fish-eating populations | Out of scope |
| 42 | Thomas HF et al. | 1996 | Cancer mortality among local authority pest control officers in England and Wales | Agricultural workers |
| 43 | Humphrey HE et al. | 1996 | Michigan's fisheater cohorts: a prospective history of exposure | Out of scope |
| 44 | Alavanja MC et al. | 1996 | The Agricultural Health Study | Protocol study |
| 45 | Bell IR et al. | 1993 | Possible time-dependent sensitization to xenobiotics: self-reported illness from chemical odors, foods, and opiate drugs in an older adult population | Out of scope |
| 46 | Wolff MS et al. | 1993 | [Blood levels of organochlorine residues and risk of breast cancer](https://pubmed.ncbi.nlm.nih.gov/8468722/) | Not dietary pesticide exposure |
